# LRP1 Receptor Controls Adipogenesis and Is Up-Regulated In Human and Mouse Obese Adipose Tissue

**DOI:** 10.1371/journal.pone.0007422

**Published:** 2009-10-12

**Authors:** Olivier Masson, Carine Chavey, Cédric Dray, Aline Meulle, Danielle Daviaud, Didier Quilliot, Catherine Muller, Philippe Valet, Emmanuelle Liaudet-Coopman

**Affiliations:** 1 IRCM, Institut de Recherche en Cancérologie de Montpellier, INSERM, U896, Université Montpellier1, CRLC Val d'Aurelle Paul Lamarque, Montpellier, France; 2 Institut National de la Santé et de la Recherche Médicale (INSERM), U858, Toulouse, France; 3 Université de Toulouse, UPS, Institut de Médecine Moléculaire de Rangueil, Equipe n°3, IFR31, Toulouse, France; 4 Institute of Pharmacology and Structural Biology CNRS UMR 5089, Université de Toulouse, Toulouse, France; 5 Service de diabétologie, Maladies métaboliques et nutrition, CHU de Nancy, Hôpital J. d'Arc, Nancy France; Karolinska Institutet, Sweden

## Abstract

The cell surface low-density lipoprotein receptor-related protein 1, LRP1, plays a major role in lipid metabolism. The question that remains open concerns the function of LRP1 in adipogenesis. Here, we show that LRP1 is highly expressed in murine preadipocytes as well as in primary culture of human adipocytes. Moreover, LRP1 remains abundantly synthesised during mouse and human adipocyte differentiation. We demonstrate that LRP1 silencing in 3T3F442A murine preadipocytes significantly inhibits the expression of PPARγ, HSL and aP2 adipocyte differentiation markers after adipogenesis induction, and leads to lipid-depleted cells. We further show that the absence of lipids in LRP1-silenced preadipocytes is not caused by lipolysis induction. In addition, we provide the first evidences that LRP1 is significantly up-regulated in obese C57BI6/J mouse adipocytes and obese human adipose tissues. Interestingly, silencing of LRP1 in fully-differentiated adipocytes also reduces cellular lipid level and is associated with an increase of basal lipolysis. However, the ability of mature adipocytes to induce lipolysis is independent of LRP1 expression. Altogether, our findings highlight the dual role of LRP1 in the control of adipogenesis and lipid homeostasis, and suggest that LRP1 may be an important therapeutic target in obesity.

## Introduction

Consumption of meals rich in fat and carbohydrates is a major causative factor of obesity, resulting in excessive white adipose tissue. Adipose tissue serves as an energy reservoir and as an endocrine organ. An increase of adipose tissue mass results from combined hypertrophy of existing adipocytes (hypertrophic adipocytes) and adipogenic differentiation of precursor cells (hyperplasic adipocytes) [1]. Expression of the nuclear peroxisome proliferator-activated receptor γ (PPARγ) is known to be crucial for the initiation of adipocyte differentiation. Indeed, mice with a targeted adipocyte-specific deletion of the PPARγ gene display a decreased adipose tissue mass [2]. Activation of PPARγ induces the expression of lipogenic genes, such as adipocyte fatty acid binding protein (aP2), and of lipolytic genes, such as hormone-sensitive lipase (HSL) [3]. Interestingly, activated PPARγ also stimulates the transcription of the low-density lipoprotein receptor-related protein 1 (LRP1) in adipocytes [4].

LRP1 is a 600-kDa multifunctional endocytic receptor that binds and internalizes a broad range of biologically diverse ligands including proteins important in lipoprotein metabolism [5]. LRP1 mediates the endocytotic internalization of dietary lipids carried in postprandial chylomicron remnants into hepatocytes by binding to Apolipoprotein E (ApoE) [6], [7], particle–bound lipoprotein lipase (LpL) [8] and hepatic lipase [9]. Interestingly, LRP1 is expressed in adipocytes [4], [10], [11] and insulin stimulation of LRP1 increases the endocytic uptake of triglycerides and cholesteryl esters from remnant lipoproteins in postprandial adipocytes in a synergistic action with lipoprotein lipase [10]. Adipose-specific LRP1-knockout mice generated by crossing LRP1*^flox/flox^* mice with *aP2-Cre* transgenic mice recently revealed its prominent role in lipid assimilation affecting energy metabolism and diet-induced obesity in mature adipocytes [12]. Even through the fundamental function of LRP1 in lipid homeostasis was recently revealed in mouse model [12], its role in adipogenesis remains to be elucidated. Here, we report that LRP1 expression is necessary for adipocyte differentiation. Silencing of LRP1 in preadipocytes by the use of siRNAs significantly inhibits the expression of PPARγ, HSL and aP2 adipocyte differentiation markers, and leads to lipid-depleted cells inept to induce lipolysis. Moreover, we corroborated the key function of LRP1 in maintaining the lipid levels in mature adipocytes. Until now, the implication of LRP1 in obesity has not been reported yet in human. Our study highlights, for the first time, that LRP1 expression is up-regulated in obese human tissue, and suggests that this receptor may be an interesting therapeutic target in obesity.

## Results

### LRP1 is highly expressed in adipocytes during adipogenesis in mouse and human

**Figure 1:**
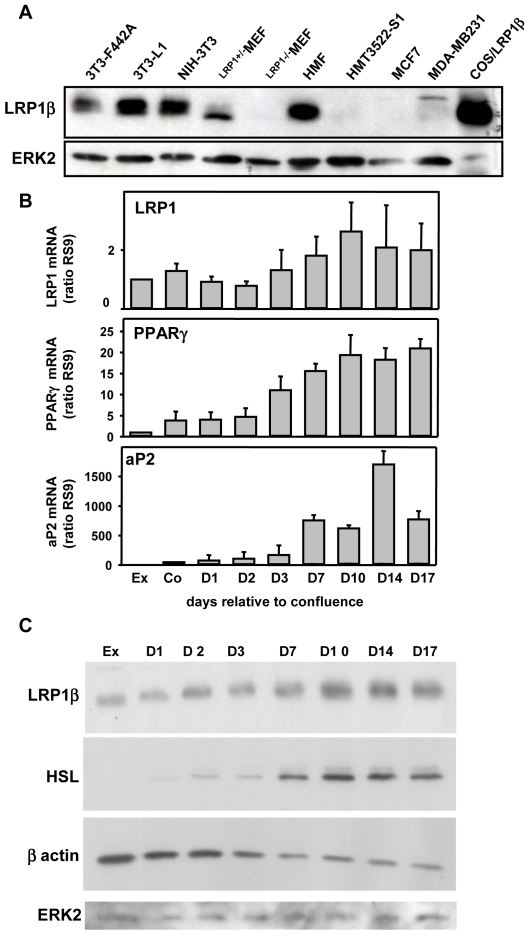
Expression of LRP1 in mouse adipocytes during adipogenesis. (A) LRP1β protein expression. Expression of LRP1β, and ERK2 were analysed by immunoblotting in preadipocytes (3T3-F442A, 3T3-L1), fibroblasts (NIH-3T3, ^LRP1−/−^MEF, ^LRP1+/−^MEF, HMF), and epithelial (HMT3522-S1, MCF7, MDA-MB-231) cells. COS cells transfected with LRP1β serve as a positive control for LRP1β expression. ERK2 was used as a loading control. (B) LRP1 mRNA expression during adipogenesis. RNA expression of LRP1, PPARγ and aP2 were analysed in exponentially growing 3T3F442A preadipocytes (Ex), in 3T3F442A grown to confluence (Co) and after the indicated time of culture in adipogenic differentiation medium by real-time quantitative RT-PCR. Mean ± SD of 5 independent experiments quantified in duplicate is shown. LRP1 mRNA expression was not statistically different during adipogenesis. (C) LRP1β protein expression during adipogenesis. Protein expression of LRP1β, HSL, βactin and ERK2 were analysed by immunoblotting in exponentially growing 3T3F442A preadipocytes (Ex) and after the indicated time following induction of the differentiation process. ERK2 was used as loading control. Similar results were observed in 3 independent experiments. LRP1β protein expression was not statistically different during adipogenesis.

In order to explore the function of adipocytic LRP1, we first investigated the level of LRP1 protein expression in preadipocytes relative to fibroblasts and epithelial cancer cells. As illustrated in Figure 1A, LRP1 was abundantly expressed in preadipocytes (3T3-F442A, 3T3-L1) and in fibroblasts (NIH-3T3, ^LRP1+/−^MEFs, HMF), when compared to epithelial mammary immortalized (HMT3522-S1) or cancer (MCF7, MDA-MB231) cells. As expected, no LRP1 expression was detected in ^LRP1−/−^MEF cells (Fig. 1A).

To assess the role of LRP1 in preadipocytes, we next examined the regulation of its expression during the course of adipogenesis in the well-established murine 3T3F442A preadipocyte cell line, which has been validated as a valuable model of adipogenesis [13] (Fig. 1B–C). LRP1 mRNA (Fig. 1B) and LRP1β protein (Fig. 1C) were expressed at high levels along adipogenesis. Even through, a tendency of LRP1 up-regulation was observed, no statistical significance was reached (Fig. 1B–C). To validate our experimental conditions, we studied in parallel the expression of PPARγ (Fig. 1B), HSL (Fig. 1C) and aP2 (Fig. 1B) adipocyte markers of differentiation. As attempted, the level of these markers was progressively increased during acquisition of the adipocyte phenotype (Fig. 1B–C). In addition, the cytoskeletal βactin protein amount was diminished during adipocyte differentiation reflecting the change in cellular morphology (Fig. 1C), as described before [14].

**Figure 2:**
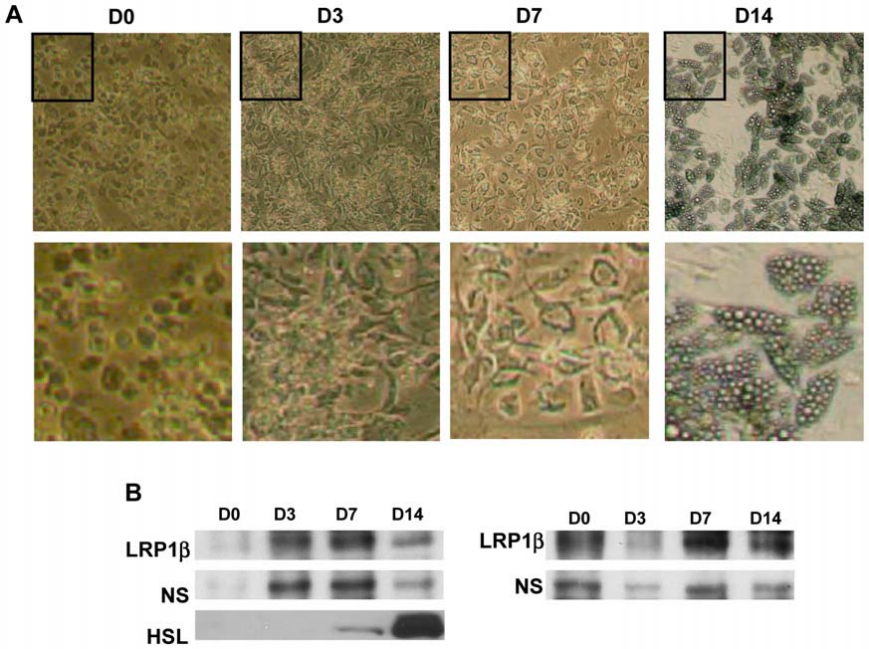
Expression of LRP1 during adipogenesis in human. (A) Micrographs of human adipocytes. Human preadipocytes isolated from subcutaneous adipose tissue digested with collagenase and separated from the stromal vascular fraction were grown for 0, 3, 7 and 14 days in the presence of the adipogenic medium as illustrated in the micrographs. A representative experiment is shown. (B) LRP1β protein expression during adipogenesis. Protein expression of LRP1β and HSL was analysed by immunoblotting after the indicated time following induction of the differentiation process. NS, non specific band showing sample loading. Two independent experiments (left and right panels) are presented.

Although 3T3F442A cells are a valuable experimental model, these preadipocytes have distinct attributes compared with human cells in primary culture beyond the obvious species differences. Therefore, we also analysed LRP1 expression during adipogenesis in human preadipocytes purified from abdominal subcutaneous adipose tissue using a recent approach [15] (Fig. 2). These primary human cells were differentiated in an efficient manner since about 75% of preadipocytes were converted to the adipocyte phenotype (Fig. 2A) and as reflected by HSL induction (Fig. 2B). As observed for the mouse adipocyte, LRP1β protein remained abundantly expressed along human adipocyte differentiation (Fig. 2B). Taken together, these findings report the presence of high LRP1 levels during adipocyte differentiation in both mouse and human.

### Silencing of LRP1 expression inhibits adipogenesis

**Figure 3:**
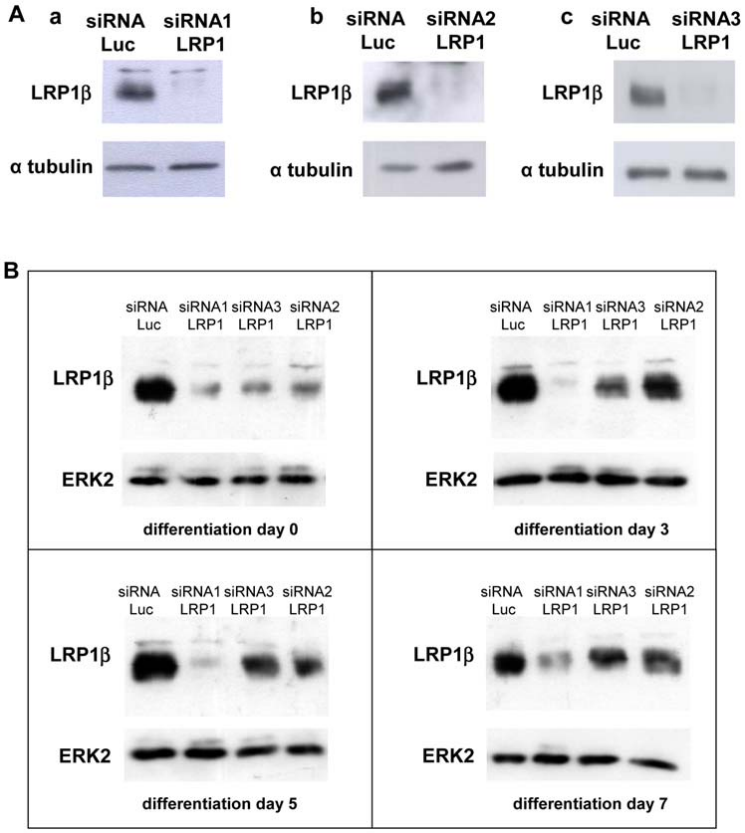
Inhibition of LRP1 expression by silencing during adipocyte differentiation. (A) Silencing of LRP1 by siRNAs. 3T3F442A preadipocytes were transiently transfected with Luc siRNA or LRP1 siRNAs. LRP1β protein expression was monitored by immunoblotting 2 days post-transfection. α tubulin was used as a loading control. A representative experiment out of 3 is shown. (B) Time-course of LRP1β protein expression after Luc siRNA or LRP1 siRNAs transfection during adipogenesis. 3T3F442A preadipocytes were transiently transfected with Luc siRNA or LRP1 siRNAs. Two-days post-transfection, LRP1β protein expression was monitored by immunoblotting 0, 3, 5 or 7 days after differentiation induction. ERK2 was used as a loading control.

**Figure 4:**
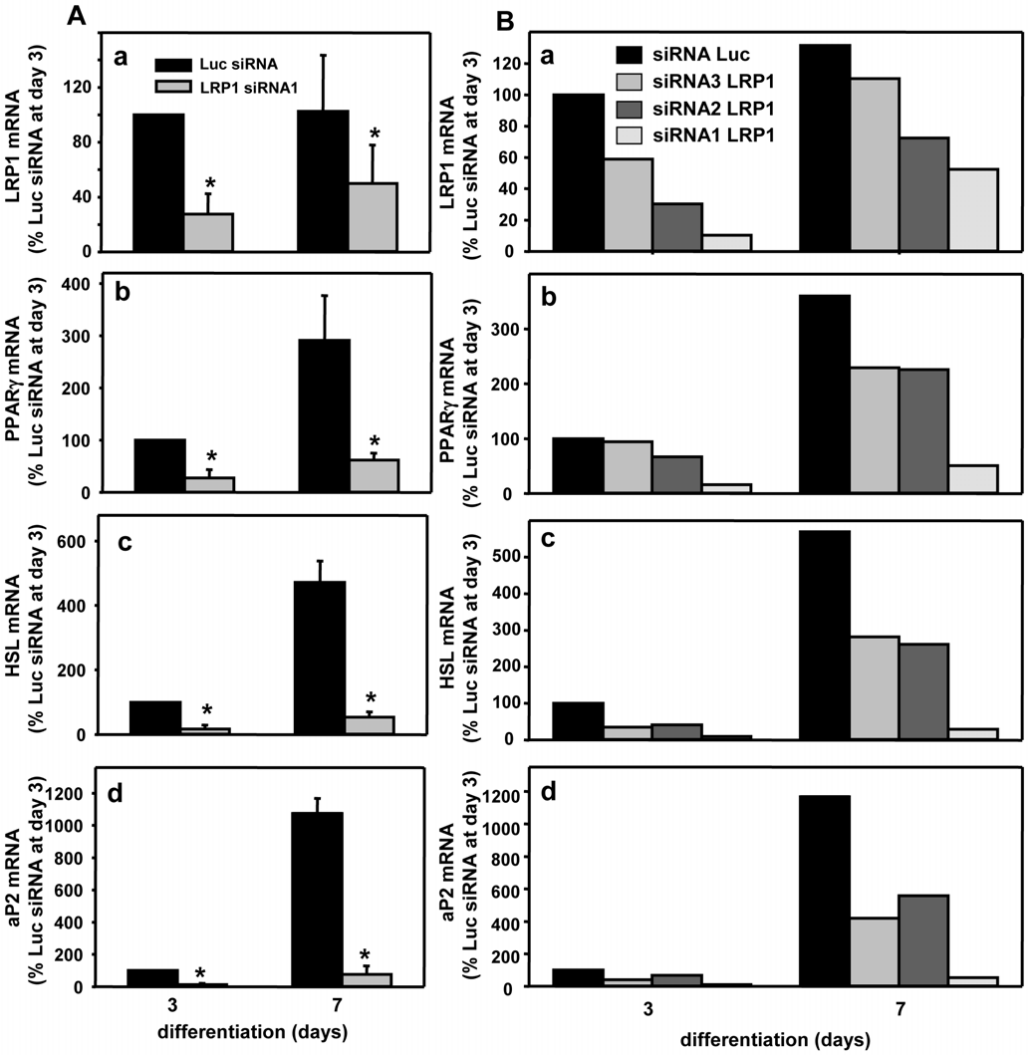
Silencing of LRP1 inhibits expression of adipocyte differentiation markers in a LRP1 dose-dependent manner. (A) mRNA expression of LRP1, PPARγ, HSL and aP2 in LRP1 siRNA1-silenced preadipocytes. RNA expression of LRP1, PPARγ, HSL and aP2 was analysed in 3T3F442A preadipocytes transfected with Luc siRNA or LRP1 siRNA1. RNA was quantified by RT-PCR in 3T3F442A cells after 3 and 7 days in adipogenic differentiation medium. Mean ± SD of 4 independent experiments is shown. *P<0.025 when compared with Luc siRNA transfected cells. (B) Inhibition of PPARγ, HSL and aP2 mRNA expression in LRP1-silenced preadipocytes in a LRP1 dose-dependent manner. RNA levels of LRP1, PPARγ, HSL and aP2 in 3T3F442A preadipocytes transfected with Luc siRNA or the 3 LRP1 siRNA1-3 were performed as described in panel A.

To determine whether preadipocyte requires LRP1 to differentiate into mature adipocyte, LRP1 expression was silenced in 3T3F442A preadipocytes using 3 LRP1 siRNAs. As shown in Figure 3A, a robust LRP1β extinction was observed two days post-transfection with LRP1 siRNAs as compared to control Luc siRNA. Consequently, we induced adipogenesis in control (Luc siRNA) and LRP1-silenced 3T3F442A preadipocytes two days post-transfection (Fig. 3B). As shown in Fig. 3B, LRP1β protein expression was inhibited by the 3 LRP1 siRNAs as compared to Luc siRNA during differentiation. The most efficient siRNA, LRP1 siRNA1, inhibited LRP1β protein still after 7 days of differentiation (Fig. 3B). A gradual lost of efficiency was observed with LRP1 siRNA2 and siRNA3 at days 3, 5 and 7 days of differentiation (Fig. 3B). Similar LRP1 silencing was observed at the mRNA level (Fig. 4A, panel a; Fig. 4B, panel a), with a significant reduction of 74% and 50% for LRP1 siRNA1 after 3 and 7 days of differentiation.

In order to evaluate the consequences of LRP1 silencing in adipocyte differentiation, we first quantified the expression of PPARγ, HSL and aP2 adipocyte differentiation markers in Luc and LRP1 siRNA transfected 3T3F442A cells (Fig. 4). LRP1 siRNA1 silencing (Fig. 4A, panel a) significantly inhibited PPARγ (Fig. 4A, panel b), HSL (Fig. 4A, panel c) and aP2 (Fig. 4A, panel d) mRNA levels at days 3 and 7 of differentiation. Extinction of LRP1 expression by LRP1 siRNA2 and siRNA3 (Fig. 4B, panel a) also reduced PPARγ (Fig. 4B, panel b), HSL (Fig. 4B, panel c) and aP2 (Fig. 4B, panel d) mRNA levels at days 3 and 7 of differentiation but to a lesser extend as compared to LRP1 siRNA1. Hence, our results indicate that the gradual lost of LRP1 expression by LRP1 siRNAs (Fig. 4B, panel a) led to a concomitant decrease of adipocyte differentiation marker expression (Fig. 4B, panels b-d). Altogether, these findings highlight that LRP1 tightly controls adipogenic expression markers in a dose-dependent manner.

**Figure 5:**
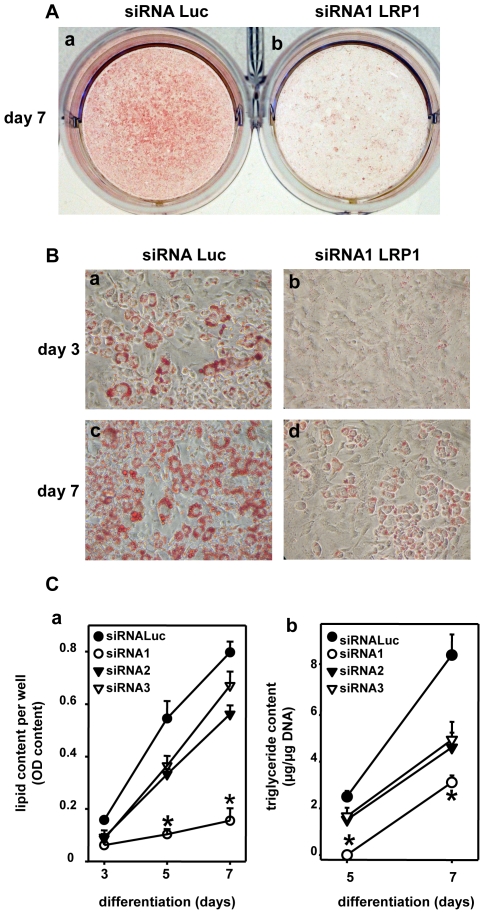
LRP1 expression is required for adipogenesis. (A) Staining of neutral lipids. Plates of 3T3F442A transfected with Luc siRNA (panel a) or LRP1 siRNA1 (panel b) at day 7 are presented. Two-days post Luc or LRP1 siRNA transfection, confluent 3T3F442A cells were grown in the presence of the adipogenic differentiation medium. After 7 days of differentiation, the extent of cellular lipid accumulation was revealed by oil Red O staining. A representative experiment out of 3 is shown. (B) Micrographs of 3T3F442A preadipocytes. Cells were transfected with Luc siRNA (panels a and c) or LRP1 siRNA1 (panels b and d) and micrographs were performed after 3 (panels a and b) and 7 (panels c and d) days of differentiation. A representative experiment out of 3 is shown. (C) Quantification of neutral lipids and triglycerides. In panel a, lipid content was quantified at 3, 5 and 7 days of differentiation in 3T3F442A transfected with Luc or LRP1 siRNAs. Mean ± SD of 3 independent experiments is shown. *P<0.0025 when compared with Luc siRNA transfected cells. In panel b, triglycerides were quantified at day 5 and 7 of differentiation in 3T3F442A transfected with Luc or LRP1 siRNAs. Results were normalised per µg of DNA. Data are presented as mean ± SD of an experiment performed in triplicate. *P<0.0025 when compared with Luc siRNA transfected cells.

Then, we analysed the cellular lipid levels in adipocytes silenced or not for LRP1 (Fig. 5). Indeed, the most obvious feature of adipocytes is the synthesis and storage of triglycerides in lipid droplets and therefore the gradual appearance and growth of lipid droplets are characteristic for adipocyte precursor cells undergoing adipogenic differentiation. The presence of these neutral lipids was detected by oil red O staining (Fig. 5A–B). As illustrated in Fig. 5A, oil red O staining after 7 days of differentiation revealed that extinction of LRP1 expression by siRNA1 strongly decreased neutral lipid droplet formation and/or accumulation. Microscopic analysis at day 3 and 7 of differentiation illustrated that, as attempted, Luc siRNA-transfected 3T3F442A cells accumulated numerous large lipid droplets and adopted a non adherent round morphology characteristic of mature adipocytes (Fig. 5B, panels a and c). By contrast, in 3T3F442A cells silenced with LRP1 siRNA1, the lipid droplet size and number were strongly reduced (Fig. 5B, panels b and d). Moreover, cells kept the morphological feature of adherent fibroblastic cells, suggesting that preadipocyte differentiation process did not occur in the absence of LRP1. Quantification of lipids afterwards demonstrated that silencing of LRP1 with LRP1 siRNA1 significantly decreased lipid content levels by 7 fold at day 7 of differentiation (Fig. 5C, panel a). LRP1 siRNA2 and siRNA3 also lowered the lipid content but to a lesser extend as compared to LRP1 siRNA1 (Fig. 5C, panel a). Similar results were obtained by quantifying triglycerides (Fig. 5C, panel b). These results reveal that LRP1 silencing in preadipocytes inhibits adipogenesis, leading to lipid-depleted cells.

**Figure 6:**
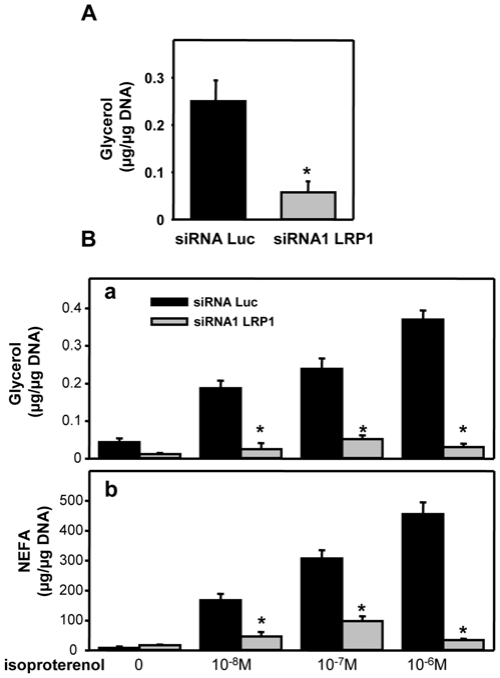
Silencing of LRP1 in pre-adipocytes inhibits lipolysis. (A) Basal lipolysis. Glycerol release in the medium after 18 h was quantified in 3T3F442A transfected with Luc or LRP1 siRNA1 after 7 days of differentiation. Results were normalised with µg of DNA. Data are presented as mean ± SD of one experiment performed in quadruplicate. P<0.025 when compared with Luc siRNA transfected cells. (B) Induced lipolysis. Glycerol and non esterified fatty acid (NAFA) releases were quantified after 7 days of differentiation in 3T3F442A cells transfected with Luc or LRP1 siRNA1 and treated with increasing concentrations of isoproterenol. Results were normalised per µg of DNA. Mean ± SD of an experiment performed in quadruplicate is presented. P<0.025 when compared with Luc siRNA transfected cells.

We finally studied the effects of silencing LRP1 in preadipocyte on lipolysis. Indeed, only mature adipocytes possess the complete apparatus for lipolysis. Our results indicating a significant decrease of HSL expression in LRP1 silenced cells (Fig. 4), we, therefore, advocate that lipolysis should be absent in LRP1-silenced preadipocytes. An alternative could be that LRP1 silenced cells have an increased lipolysis, leading to lipid depleted cells. However, our results revealed that glycerol release over 18 h was significantly reduced in LRP1 silenced cells, indicating inhibition of basal lipolysis (Fig. 6A). Moreover, glycerol and NEFA released in the media after isoproterenol treatment were also significantly decreased in LRP1 silenced cells, evidencing inhibition of induced-lipolysis. Therefore, extinction of LRP1 in preadipocytes abolishes expression of adipocyte differentiation markers and leads to lipid-depleted cells inept to induce lipolysis.

### LRP1 expression is up-regulated in human and mouse obese adipose tissues

**Figure 7:**
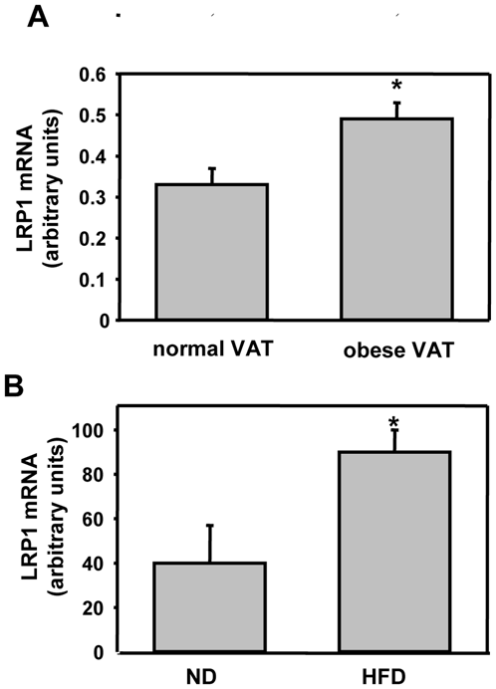
LRP1 expression is up-regulated in human and mouse obese adipose tissues. (A) LRP1 expression in adipose tissue from lean and obese human. LRP1 mRNA level was quantified in human intra-abdominal visceral adipose tissue samples (VAT) obtained from 27 morbide grade III obese patients (44.5+/−1.8 year old, BMI: 47.6 +/− 1.3 kg/m^2^) before a bariatric surgery and from 10 control patients undergoing abdominal lipectomy for plastic surgery (42.7 +/− 4.5 year old, BMI: 23.1 +/− 3.3 kg/m^2^). Results are mean values +/− SEM, *P<0.01 when compared with controls (normal VAT). (B) LRP1 expression in adipocytes from obese mouse. LRP1 mRNA level was quantified in adipocytes isolated from intra-abdominal adipose tissues from 30-week-old overweight C57Bl6/J mice fed in high-fat diet (HFD) and from C57Bl6/J control mice fed in normal diet (ND). Results are mean value +/− SEM from 5 mice for ND group and 4 mice for HFD group. *P<0.05 when compared with ND controls.

Obesity is characterized by the increase of intracellular lipid accumulation which shows a significant correlation with adipocyte differentiation. LRP1 mRNA expression was investigated in human intra-abdominal visceral adipose tissue (VAT) from lean (42.7±4.5 year old, BMI: 23.1±3.3 kg/m^2^) and obese (44.5±1.8 year old, BMI: 47.6±1.3 kg/m^2^) human (Fig. 7). Interestingly, LRP1 mRNA was significantly increased in human obese adipose tissue (Fig. 7A). To assess whether this LRP1 up-regulation was a general characteristic of obese adipocytes, we then analysed LRP1 mRNA expression in adipocytes isolated from C57BI6/J mice either fed a HFD or ND (Fig. 7B). As attempted HFD-fed C57BI6/J mice exhibited a significant increase in body mass (47.6±1.4 g) when compared to their control littermates (31.1±1.2 g) (data not shown). LRP1 expression was significantly enhanced in adipocytes of HFD obese mice when compared to ND control mice (Fig. 7B). Altogether, our results indicate that LRP1 expression is up-regulated in human and mouse obese adipose tissues.

### Silencing of LRP1 expression inhibits the cellular lipid content of fully-differentiated adipocytes

**Figure 8:**
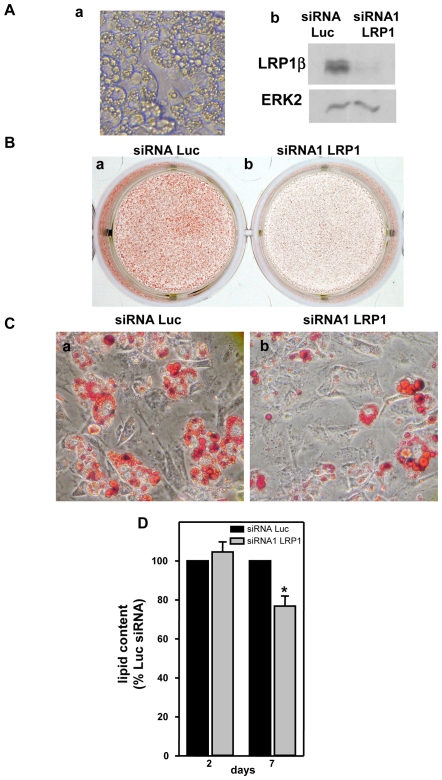
LRP1 silencing in fully-differentiated adipocytes leads to lipid-depleted cells. (A) Silencing of LRP1 in fully-differentiated adipocyte. 3T3F442A pre-adipocytes were maintained for 10 days in adipogenic differentiation medium (panel a) and were transfected with Luc siRNA or LRP1 siRNA1. LRP1β protein expression was monitored by immunoblotting 2 days post-transfection (panel b). ERK2 was used as a loading control. (B) Staining of neutral lipids. Plates of differentiated 3T3F442A transfected with Luc siRNA (panel a) or LRP1 siRNA1 (panel b) are shown. After siRNA transfection, 3T3F442A mature adipocytes were grown in DMEM + 10% FCS. The extent of cellular lipid accumulation was determined at day 2 and 7 of culture by oil Red O staining. A representative experiment out of 3 is shown. (C) Micrographs of 3T3F442A adipocytes. Micrographs of differentiated adipocytes transfected with Luc siRNA (panel a) or LRP1 siRNA1 (panel b) were performed 7 days post-transfection. A representative experiment out of 3 is shown. (D) Quantification of lipid content. Lipid content was quantified at days 2 and 7 post-tranfection of differentiated adipocytes transfected with Luc or LRP1 siRNA1. Mean ± SD of 3 independent experiments is shown. *P<0.005 when compared with Luc siRNA transfected cells.

**Figure 9:**
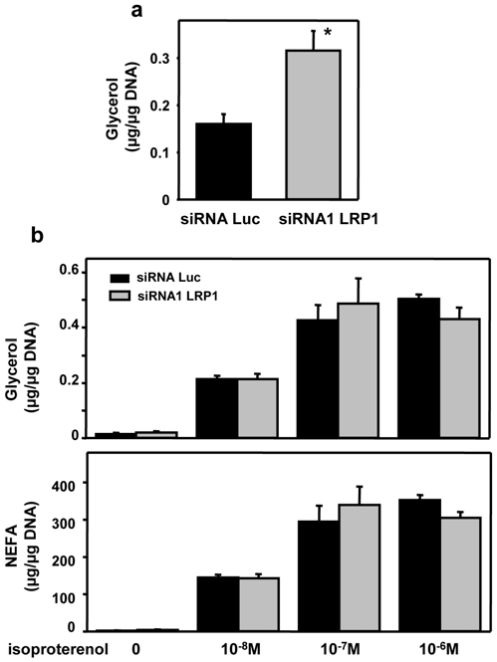
Analysis of lipolysis in fully-differentiated adipocytes silenced for LRP1. Adipocytes differentiated for 10 days were transfected with Luc siRNA or LRP1 siRNA1. Two days post-transfection, glycerol release over 18 h (panel a) and glycerol and NEFA release after isoproterenol stimulation (panel b) were quantified. Results were normalised per µg of DNA. Mean ± SD of an experiment in quadruplicate is shown. P<0.025.

Since we observed that LRP1 expression was up-regulated in obese adipose tissues, we finally investigated whether extinction of LRP1 expression in fully-differentiated adipocytes could diminish their lipid content (Fig. 8), as recently suggested in adipose-specific LRP1−/− mouse model [12]. 3T3F442A preadipocytes were cultured for 10 days in adipogenic differentiation medium (Fig. 8A, panel a) and fully-differentiated adipocytes were transiently transfected with Luc siRNA or LRP1 siRNA1 (Fig. 8A, panel b). Oil red O staining revealed that, after 7 days of culture, the lipid droplet size and number were decreased in LRP1 siRNA1 transfected adipocytes (Fig. 8B–C). Quantification of lipids at days 2 and 7 of culture revealed that, in Luc siRNA transfected adipocytes, the level of cellular lipid remained unchanged whereas, in LRP1 siRNA1 transfected cells, the amount of lipid was significantly diminished by 27.8% (Fig. 8D). Interestingly, basal lipolysis was significantly stimulated in LRP1 siRNA1 transfected adipocytes (Fig. 9, panel a). We postulate that, since cells could not internalized triglycerides in the absence of LRP1 as previously shown [12], they were metabolizing their intracellular lipid stock. However, induced lipolysis was not different in LRP1 and Luc siRNA transfected cells (Fig. 9, panel b). Altogether, these findings highlight, for the first time, the crucial role of LRP1 in controlling adipogenesis and maintaining the lipid content in fully-differentiated adipocytes, and suggest that LRP1 may be an important therapeutic target in obesity.

## Discussion

Here, we show that LRP1 is highly expressed in 3T3F442A murine preadipocyte cell line, as previously described for 3T3L1 preadipocytes and embryonic fibroblasts [16], [17]. Since LRP1 is expressed at high levels in preadipocytes, it therefore can be involved in the early steps of adipocyte differentiation. Indeed, our report demonstrate that LRP1 silencing by siRNAs in 3T3F442A preadipocytes significantly inhibits the expression of PPARγ, HSL and aP2 adipocyte markers of differentiation and leads to lipid-depleted cells, indicating that LRP1 is a key regulator of the adipogenic process. Our experiments using 3 LRP1 siRNAs show that LRP1 extinction abrogates adipocyte differentiation in a LRP1 dose-dependent manner. Our results also pinpoint that extinction of LRP1 expression in pre-adipocytes prevents their lipolytic response both at a basal level and after isoproterenol induction. Therefore the inhibition of lipid accumulation and triglyceride levels observed in LRP1-silenced preadipocytes is not the consequence of a stimulation of lipolysis, indicating a direct effect of LRP1 on the control of the adipogenic process. PPARγ is known to be crucial for the initiation of adipocyte differention [2]. Interestingly, PPARγ has been described to induce LRP1 transcription [4] and our results indicate that LRP1 silencing inhibits PPARγ expression. This suggests the existence of a regulatory loop between these two factors. Our findings reveal that LRP1 silencing inhibits the expression of PPARγ by 80% at day 3 of differentiation, indicating an early effect of LRP1 at the commencement of adipogenesis. It is therefore important to determine how LRP1 lost down-regulates PPARγ expression. A recent study demonstrates that LRP1 controls gene transcription *via* Regulated Intramembrane Proteolysis through the cytoplasmic release of LRP1 intracellular domain, LRP1-ICD [18]. Studies in the future will investigate whether LRP1-ICD regulates PPARγ expression in preadipocytes.

Our results also indicate, for the first time, that LRP1 expression is up-regulated in human obese tissue. This up-regulation of LRP1 expression in the VAT of overweight/obese patients is in consensus with our results in obese mice. Obesity is characterized by the increase of intracellular lipid accumulation which shows a significant correlation with adipocyte differentiation. Terminally differentiated adipocytes cannot divide. Hence, alterations in the number of fat cells within the body must be accomplished by the differentiation of preadipocytes, which act as a renewable source of adipocytes. Interestingly, our *in vitro* study in 3T3F442A cells indicates that LRP1 expression is required for adipocyte differentiation and since LRP1 is abundantly expressed in adipocytes, we propose that LRP1 may participate in the onset of obesity. The crucial role of LRP1 in obesity is also supported by a recent study showing that adipocyte LRP1−/− mice have an overall decrease in fat mass and are protected from high-fat diet-induced obesity [12]. Our experiments further show that silencing of LRP1 in fully-differentiated adipocytes significantly reduces cellular lipid content, suggesting that LRP1 may be an important therapeutic target in obesity. Most functional studies on adipocyte differentiation and function have been performed in the murine adipogenic 3T3L1 cell line and in genetically modified mice. However, there are fundamental differences in the lipoprotein metabolism of mouse and human [19]. Therefore, it is important to investigate the LRP1 expression in human adipocytes as presented in this report.

Results from the literature have suggested that LRP1 is a likely contributor to adipogenesis, adipocyte homeostasis and obesity given its high expression in adipocytes [4], [10], [11], that it binds ApoE [7], [8], LpL [20], [21] and hepatic lipase [9], and that it collaborates with heparin sulfate proteoglycans to mediate the internalization of ApoE and LpL [22], [23]. The dietary lipids are carried in chylomicron remnants (CR) which are taken up into the liver mainly *via* LRP1. LRP1 interacts with CR *via* ApoE [6], [7] and LpL *in vitro* and *in vivo* [8], [24]. Interestingly, ApoE has been reported to be crucial for the accumulation of triglycerides in mouse adipocytes [25] and also appears to have an important function in adipocyte differentiation [26] and obesity [27]. Altogether, these observations strongly suggest that a deregulation of LRP1 expression may have important consequences in adipocytes and obesity.

Some insight into LRP1 function in mature adipocyte was obtained by generating mice with adipocyte-specific inactivation of the LRP1 gene [12]. Adipocyte LRP1 knockout mice displayed delayed postprandial lipid clearance, smaller fat stores, and lipid-depleted adipocytes which resulted in reduced body weight due to overall decrease in fat mass. This work highlights the importance of adipocyte LRP1 in postprandial triglyceride metabolism, where LRP1 in collaboration with LpL mediates both the endocytic and lipolytic processes responsible for triglyceride catabolism [23], [28], [29]. Our results are in accordance with this study and indicate that extinction of LRP1 expression in mature adipocytes leads to increased basal lipolysis. In addition, we show that the capacity of mature adipocytes to induce lipolysis in response to isoproterenol is not modified by LRP1 silencing. This suggests that LRP1 does not directly control lipolysis. We propose that inhibition of LRP1 expression stimulates basal lipolysis in order to compensate the lack of lipid intake in the absence of LRP1. Recently, it was shown that LRP1 is also required for lipolysis and the control of intracellular cholesterol storage and fatty acid synthesis *via* the Wnt5a signaling pathway in ^LRP1−/−^MEF cells [30].

In conclusion, our findings highlight that LRP1 plays a crucial role in the control of adipogenesis and lipid homeostasis in mature adipocytes. Moreover, our results indicate that LRP1 is up-regulated in human obese tissues and mouse adipocytes. Therefore, we propose that LRP1 targeting in obesity may lead to a reduction of hypertrophic and hyperplasic adipocytes.

## Materials and Methods

### Ethics Statement

All subjects gave their informed written consent to participate to the study, and investigations were performed in accordance with the declaration of Helsinki as revised in 2000 (http://www.wma.net/e/policy/b3.htm).

### Cells and cell culture

Cell lines were cultured in DMEM (Invitrogen) supplemented with 10% fetal calf serum (FCS). Differentiation was induced by incubating 3T3F442A confluent cells in differentiation medium (DMEM supplemented with 10% FCS and 50 nM insulin) as described previously for up to 7 days [31]. After 7 days of differentiation, cells were maintained in DMEM with 10% FCS.

### Human samples

Human adipose tissue was collected according to the guidelines of the Ethical Committee of Toulouse-Rangueil and Nancy J. d'Arc Hospitals and received full ethical approval from the Ethical Committee of Toulouse-Rangueil and Nancy J. d'Arc Hospitals. All subjects gave their informed written consent to participate to the study. Human abdominal visceral (VAT) adipose tissue samples were obtained from 10 patients healthy volunteers (42.7 +/− 4.5 yr old, BMI: 23.1 +/− 3.3 kg/m^2^) undergoing abdominal lipectomy for plastic surgery. No clinical data from these patients were available. Human abdominal visceral adipose tissue samples were obtained from 27 morbidly (grade III) obese subjects (44.5+/−1.8 yr old, BMI: 47.6 +/− 1.3 kg/m^2^) before a bariatric surgery. All subjects were drug-free and besides obesity they did not suffer of any disease. Tissue samples were immediately frozen in liquid nitrogen and stored at −80°C. Total RNAs of isolated adipocytes were extracted and LRP1 expression analysed by RT-PCR. For *in vitro* differentiation, human preadipocytes were isolated from human subcutaneous adipose tissue obtained from patients undergoing abdominal lipectomy at the plastic surgery department of Rangueil Hospital (Toulouse, France) under the agreement of local ethic committee. All subjects gave their informed written consent to participate to the study. Adipose tissue pieces were immediately used for collagenase digestion as previously described [15]. The digestate was centrifuged to separate adipocytes from the stroma-vascular fraction, containing preadipocytes (pellet). Cells isolated from the SVF fraction were induced to differentiate into adipocytes as previously described [31]. Briefly, confluent cells (day 0) were induced to differentiate in DMEM/Ham's F12 (1∶1) medium containing 0.01 mg/ml transferrin, 100 nM cortisol, 0.2 nM triiodothyronine, and 20 nM insulin. To trigger differentiation, 25 nM dexamethasone, 500 mM IBMX and 2 mM rosiglitazone were present from day 0 to day 4. Intracellular accumulation of lipid droplets became clearly evident at day 10 [15].

### Mice

Mice were handled in accordance with the principles and guidelines established by the National Institute of Medical Research (INSERM). C57Bl6/J female mice were obtained from Charles River laboratory (l'Arbresle, France). Mice were housed conventionally in a constant temperature (20–22°C) and humidity (50–60%) animal room and with a 12 h light–dark cycle. All mice had free access to food and water throughout the experiment. C57Bl6/J mice were assigned to normal-fat diet (ND) or high-fat diet (HFD) (SAFE, France). Energy contents of the specific diets were (% kcals): 20% protein, 70% carbohydrate, and 10% fat for ND; 20% protein, 35% carbohydrate, and 45% fat for HFD. The main source of fat in HFD was lard (20 g/100 g of food). C57Bl6/J (10 week old) mice were fed a ND or HFD for 20 weeks. All mice were sacrificed at 30 weeks of age.

### Isolation of adipocytes from mouse adipose tissue

Mouse intra-abdominal adipose tissues were dissected immediately after sacrifice, minced in 5 ml of Dulbecco's modified Eagle's medium (DMEM; Life Technologies, Inc., Invitrogen, Paisley, UK) supplemented with 1 mg/ml collagenase (SIGMA) and 1% BSA for 30 min at 37°C under shaking. Digestion was followed by filtration through a 150 µm screen, and the floating adipocytes were separated from the medium containing the stroma-vascular fraction (SVF). Adipocytes were washed twice in DMEM and further processed for RNA extraction using the RNeasy mini kit (Qiagen, Germany).

### Immunoblots

Cells were lysed in lysis buffer (50 mM HEPES pH 7.5, 150 mM NaCl, 10% glycerol, 1% Triton X100, 1.5 mM MgCl_2_, 1 mM EGTA, 100 mM NaF, 10 mM NaPPI, 500 µm Na-Vanadate, 1 mM PMSF, 10 µM Aprotinine, and a protease inhibitor cocktail). After gentle shaking for 20 min at 4°C, cell extracts were obtained by centrifugation in a microfuge at 13,000 rpm for 15 min at 4°C. Equal amounts of protein (100 µg) from cell extracts, quantitated by the Bradford assay, were separated on a 7% gel by SDS-PAGE. Proteins were electro-transferred to PVDF membrane and incubated with 1 µg/ml anti-α tubulin (NeoMarkers), 1 µg/ml anti-β−actin (Sigma), 1 µg/ml ERK2 (D-2, Santa Cruz Biotechnology), 11H4 anti-LRP1β hybridoma (1/10 dilution, ATCC) or 0.4 µg/ml anti-HSL (Santa Cruz). Proteins were then visualized with horseradish peroxidase-conjugated sheep anti-mouse immunoglobulin (ECL Amersham) or horseradish peroxidase-conjugated donkey anti-rabbit immunoglobulin (ECL Amersham) followed by the Renaissance chemiluminescence system (Perkin Life Sciences).

### RNA extraction and analysis

Total RNA was extracted using the RNeasy minikit (QIAGEN Sciences, Maryland) according to the manufacturer's instructions. Reverse transcription of total RNA was performed at 37°C using Moloney murine leukemia virus reverse transcriptase enzyme (Invitrogen, Carlsbad, CA) and random hexanucleotide primers (Promega, Madison, WI). Quantitative PCR was carried out by real-time PCR using a LightCycler and the DNA double-strand-specific SYBR green I dye for detection (Roche, Basel, Switzerland). Results were normalized to RS9 levels. Sequences of primers are:

mouse RS9 (sens 5′CGGCCCGGGAGCTGTTGACG3′, reverse 5′CTGCTTGCGGACCCTAATGTGACG3′),

mouse aP2 (sens 5′AACACCGAGATTTCCTTCAA3′, reverse 5′AGTCACGCCTTTCATAACACA3′),

mouse LRP1 (sens 5′GACCAGGTGTTGGACACAGATG3′, reverse 5′AGTCGTTGTCTCCGTCACACTTC3′),

mouse HSL (sens 5′CTGAAGGCTCTGAGTTGGTCAA3′, reverse 5′GGCTTACTGGGCACAGATACCT3′),

mouse PPARγ (sens 5′ AGGCCGAGAAGGAGAAGCTGTTG3′, reverse 5′TGGCCACCTCTTTGCTCTGCTC3′),

human LRP1 (sens 5′TAGACCGGCCCCCTGTGCTGTTGA3′, reverse 5′GGTCTGCCGCGTGCTCGTAGGTGT3′).

### siRNAs and 3T3F442A transfection

Silencing of LRP1 gene expression in adipocytes was achieved by the use of siRNAs. Duplexes of 21-nucleotide mouse LRP1 siRNA1 (target sequence AAGCATCTCAGTAGACTATCA) [32], mouse LRP1 siRNA2 (target sequence AACTTCTTAAACTCATAGCTT) (Dharmacon), mouse LRP1 siRNA3 (target sequence AAGCAGTTTGCCTGCAGAGAC) or firefly luciferase (Luc) siRNA (target sequence AACGTACGCGGAATACTTCGA) were synthesized by MWG Biotech S.A. (France). 2 10^6^ 3T3F442A mature adipocytes were transiently transfected with 2 µg of siRNA using Nucleofector Technology (Amaxa biosystems) according to the manufacturer's instructions using kit L (# VCA-1005) and were replated in 2 wells of 6-well plates. After 48 h of transfection, cells have recovered and were maintained in DEM 10% FCS for further analysis. Analysis of LDH release monitored at 48 h revealed no toxicity of transfecting mature adipocytes with LRP1 or Luc siRNAs (CytoTox96 Non-Radioactive Cytotoxicity Assay, Promega). For preadipocytes, 10^6^ cells were transfected and were treated with insulin 48 h post-transfection.

### Oil Red O staining

3T3F442A adipocytes were washed with phosphate-buffered saline (pH 7.4) and then fixed with Antigenfix (Diapath, Italy). Cells were stained with Oil Red O dye (saturated Oil Red O dye in six parts of isopropanol and four parts of water), an indicator of cell lipid content, and then exhaustively rinsed with water. Spectrophotometric quantification of lipids was performed by dissolving the stained oil droplets with isopropanol and measuring absorbance at 540 nm as previously described [33].

### Quantification of triglycerides

Cells scraped in PBS were centrifugated for 5 min at 1200 rpm at 4°C. Cell pellet was dissolved in 40 µl isopropanol and centrifugated at 13 000 rpm for 5 min at 4°C. The supernatant was used to determine the triglyceride content using the Triglyceride FS kit (Diasys Diagnostic Systems, Germany) according to the manufacturer's instructions. The pellet was used to quantify DNA concentration by a diaminobenzoic aci fluorescence assay [34].

### Lipolysis Assay

Cells were incubated in DMEM without serum in the presence of 2% fatty acid-free bovine serum albumin (A7030, Sigma) overnight. Fresh medium containing isoproterenol was added for 90 min. Glycerol and NEFA contents in the medium were measured with free glycerol reagent (F6428 Sigma) and NEFA C kit (Wako Chemicals, Germany).

### Statistical analysis

Results are expressed as means ± SEM. Statistical differences between two groups were evaluated using Student's *t* tests. The level of significance was set at *P*<0.05.
